# Supplementary material for: High Fruit and Vegetable Consumption and Moderate Fat Intake Are Associated with Higher Carotenoid Concentration in Human Plasma
Source: Antioxidants (Basel). 2021 Mar 17;10(3):473. doi: 10.3390/antiox10030473 (PMC8002704; doi:10.3390/antiox10030473)
Supplement: Supplementary file 1 [file antioxidants-10-00473-s001.pdf]

antioxidants

MDPI

Article

Supplementary Materials: High Fruit and Vegetable Consumption and Moderate Fat Intake Are Associated with Higher Carotenoid Concentration in Human Plasma

María Marhuenda-Muñoz [1,2], José Fernando Rinaldi de Alvarenga [3], Álvaro Hernáez [1,4,5,6], Anna Tresserra-Rimbau [1,2], Miguel Ángel Martínez-González [1,7,8], Jordi Salas-Salvadó [1,9,10,11], Dolores Corella [1,12], Mireia Malcampo [13], José Alfredo Martínez [1,14,15], Ángel M. Alonso-Gómez [1,16,17], Julia Wärnberg [1,18], Jesús Vioque [19,20], Dora Romaguera [1,21], José López-Miranda [1,22], Ramón Estruch [1,23], Francisco J. Tinahones [1,24], José Lapetra [1,25], J. Lluís Serra-Majem [1,26], Aurora Bueno-Cavanillas [18,27], Josep A. Tur [1,21,28], Vicente Martín Sánchez [15,29], Xavier Pintó [1,30], Miguel Delgado-Rodríguez [15,31], Pilar Matía-Martín [32], Josep Vidal [33,34], Clotilde Vázquez [1,35], Lidia Daimiel [15], Emilio Ros [1,36], Mercè Serra-Mir [36], Zenaida Vázquez-Ruiz [1,7], Stephanie K. Nishi [1,9,10,11], Jose V. Sorlí [1,12], María Dolores Zomeño [5,13,], María Angeles Zulet [1,14,15], Jessica Vaquero-Luna [16,17], Rosa Carabaño-Moral [37], Leyre Notario-Barandiaran [19,20], Marga Morey [1,21], Antonio García-Ríos [1,22], Ana M. Gómez-Pérez [1,24], José Manuel Santos-Lozano [1,25], Pilar Buil-Cosiales [1,7,38], Josep Basora [1,9,11,39], Olga Portolés [1,12], Helmut Schröder [13,19], Itziar Abete [1,14,15], Itziar Salaverria-Lete [16], Estefanía Toledo [1,7], Nancy Babio [1,9,10,11], Montse Fitó [1,13], Miriam Martínez-Huélamo [2] and Rosa M Lamuela-Raventós [1,2,*]

1 Centro de Investigación Biomédica en Red Fisiopatología de la Obesidad y la Nutrición (CIBEROBN), Instituto de Salud Carlos III, 28029 Madrid, Spain; mmarhuendam@ub.edu (M.M.-M.); alvaro.hernaez@fhi.no (Á.H.); annatresserra@ub.edu (A.T.-R.); mamartinez@unav.es (M.Á.M.-G.); jordi.salas@urv.cat (J.S.-S.); dolores.corella@uv.es (D.C.); jalfmtz@unav.es (J.A.M.); angelmago13@gmail.com (Á.M.A.-G.); jwarnberg@uma.es (J.W.); mariaadoracion.romaguera@ssib.es (D.R.); jlopezmir@uco.es (J.L.-M.); restruch@clinic.cat (R.E.); fjtinahones@hotmail.com (F.J.T.); joselapetra543@gmail.com (J.L.); lserra@dcc.ulpgc.es (J.L.S.-M.); pep.tur@uib.es (J.A.T.); xpinto@bellvitgehospital.cat (X.P.); clotilde.vazquez@fjd.es (C.V.); eros@clinic.cat (E.R.); zvazquez@unav.es (Z.V.-R.); stephanie.nishi@urv.cat (S.K.N.); jose.sorli@uv.es (J.V.S.); mazulet@unav.es (M.A.Z.); marga.morey@yahoo.es (M.M.); angarios2004@yahoo.es (A.G.-R.); anamgp86@gmail.com (A.M.G.-P.); jsantos11@us.es (J.M.S.-L.); pilarbuilc@gmail.com (P.B.-C.); jbasora@idiapjgol.org (J.B.); olga.portoles@uv.es (O.P.); iabetego@unav.es (I.A.); etoledo@unav.es (E.T.); nancy.babio@urv.cat (N.B.); mfito@imim.es (M.F.)
2 Department of Nutrition, Food Science and Gastronomy, School of Pharmacy and Food Sciences and XaRTA, Institute of Nutrition and Food Safety (INSA-UB), University of Barcelona, 08921 Santa Coloma de Gramenet, Spain; mmartinezh8@gmail.com
3 Food Research Center (FoRC), Department of Food Science and Experimental Nutrition, School of Pharmaceutical Sciences, University of São Paulo, 05508-000 São Paulo, Brazil; zehfernando@gmail.com
4 Centre for Fertility and Health, Norwegian Institute of Public Health, 0473 Oslo, Norway
5 Blanquerna School of Health Sciences. Universitat Ramon Llull, 08025 Barcelona, Spain; mzomeno@imim.es
6 August Pi Sunyer Biomedical Research Center (IDIBAPS), 08036 Barcelona, Spain
7 Department of Preventive Medicine and Public Health, University of Navarra, IdiSNA, 31008 Pamplona, Spain
8 Department of Nutrition, Harvard T.H. Chan School of Public Health, Boston, MA 02115, USA
9 Departament de Bioquímica i Biotecnologia, Unitat de Nutrició, Universitat Rovira i Virgili, 43204 Reus, Spain
10 Nutrition Unit, University Hospital of Sant Joan de Reus, 43201 Reus, Spain
11 Institut d'Investigació Sanitària Pere Virgili (IISPV), 43201 Reus, Spain
12 Department of Preventive Medicine, University of Valencia, 46010 Valencia, Spain
13 Unit of Cardiovascular Risk and Nutrition, Institut Hospital del Mar de Investigaciones Médicas (IMIM), 08007 Barcelona, Spain; mireiamalcampo@gmail.com (M.M.); HSchoeder@imim.es (H.S.)
14 Center for Nutrition Research, Department of Nutrition, Food Sciences, and Physiology, University of Navarra, 31008 Pamplona, Spain
15 Precision Nutrition Program, IMDEA Food, CEI UAM + CSIC, 28049 Madrid, Spain; vicente.martin@unileon.es (V.M.S.); mdelgado@ujaen.es (M.D.-R.); lidia.daimiel@imdea.org (L.D.)

[16] Bioaraba Health Research Institute, Cardiovascular, Respiratory and Metabolic Area, 01009 Vitoria-Gasteiz, Spain; luna_jess_@hotmail.com (J.V.-L.); itziar_salaverria@yahoo.es (I.S.-L.)
[17] Osakidetza Basque Health Service, Araba University Hospital, University of the Basque Country UPV/EHU, 01009 Vitoria-Gasteiz, Spain
[18] Department of Nursing, School of Health Sciences, Instituto de Investigación Biomédica de Málaga (IBIMA), University of Málaga, 29010 Málaga, Spain; abueno@ugr.es
[19] CIBER de Epidemiología y Salud Pública (CIBERESP), Instituto de Salud Carlos III, 28029 Madrid, Spain; vioque@umh.es (J.V.); lnotario@umh.es (L.N.-B.)
[20] Unit of Nutritional Epidemiology, Miguel Hernandez University, ISABIAL-FISABIO, 03010 Alicante, Spain
[21] Health Research Institute of the Balearic Islands (IdISBa), 07120 Palma de Mallorca, Spain
[22] Department of Internal Medicine, Maimonides Biomedical Research Institute of Cordoba (IMIBIC), Reina Sofia University Hospital, University of Cordoba, 14004 Cordoba, Spain
[23] Internal Medicine Service, Hospital Clínic, University of Barcelona, 08036 Barcelona, Spain
[24] Department of Endocrinology, Virgen de la Victoria Hospital, Instituto de Investigación Biomédica de Málaga (IBIMA), University of Málaga, 29010 Málaga, Spain
[25] Research Unit, Department of Family Medicine, Distrito Sanitario Atención Primaria Sevilla, 41010 Sevilla, Spain
[26] Research Institute of Biomedical and Health Sciences (IUIBS), University of Las Palmas de Gran Canaria & Centro Hospitalario Universitario Insular Materno Infantil (CHUIMI), Canarian Health Service, 35016 Las Palmas de Gran Canaria, Spain
[27] Department of Preventive Medicine and Public Health, University of Granada, 18016 Granada, Spain
[28] Research Group on Community Nutrition & Oxidative Stress, IUNICS, University of Balearic Islands, 07122 Palma de Mallorca, Spain
[29] Institute of Biomedicine (IBIOMED), University of León, 24071 León, Spain
[30] Lipids and Vascular Risk Unit, Internal Medicine, Hospital Universitario de Bellvitge, Hospitalet de Llobregat, 08908 Barcelona Spain
[31] Division of Preventive Medicine, Faculty of Medicine, University of Jaén, 23071 Jaén, Spain
[32] Department of Endocrinology and Nutrition, Instituto de Investigación Sanitaria Hospital Clínico San Carlos (IdISSC), 28040 Madrid, Spain; pilar.matia@gmail.com
[33] CIBER Diabetes y Enfermedades Metabólicas (CIBERDEM), Instituto de Salud Carlos III (ISCIII), 28029 Madrid, Spain; jovidal@clinic.cat
[34] Department of Endocrinology, Institut d'Investigacions Biomédiques August Pi Sunyer (IDIBAPS), Hospital Clinic, University of Barcelona, 08036 Barcelona, Spain
[35] Department of Endocrinology and Nutrition, Hospital Fundación Jimenez Díaz, Instituto de Investigaciones Biomédicas IISFJD, University Autonoma, 28040 Madrid, Spain
[36] Department of Endocrinology and Nutrition, Hospital Clínic, 08036 Barcelona, Spain; SERRAMIR@clinic.cat
[37] Unidad de Gestión Clínica Arroyo de la Miel, Distrito de Atención Primaria Costa del Sol, Servicio Andaluz de Salud, 29630 Benalmádena, Málaga, Spain; rosa.carabano.sspa@juntadeandalucia.es
[38] Osasunbidea, Servicio Navarro de Salud, Atención Primaria, 31003 Pamplona, Spain
[39] IDIAP Jordi Gol i Gurina, 43202 Reus, Spain
* Correspondence: lamuela@ub.edu; Tel.: +34-934034843

Table S1. Limits of detection and quantification, concentration range and r of the calibration curves of the different carotenoids (μmol/L).

Analyte	LoD	LoQ	Concentration range	r
astaxanthin	0.009	0.17	0.17 – 5.03	0.9965
lutein	0.023	0.88	0.88 – 8.79	0.9978
zeaxanthin	0.120	0.88	0.88 – 5.27	0.9930
canthaxanthin	0.0005	0.089	0.089 – 5.31	0.9975
E-*β*-apo-8′-carotenal	0.018	0.12	0.12 – 7.20	0.9967
β-cryptoxanthin	0.004	0.02	0.02 – 18.09	0.9982
13-*Z*-*β*-carotene	0.072	0.93	0.93 – 18.63	0.9909
α-carotene	0.037	0.12	0.12 – 1.86	0.9932
β-carotene	0.021	0.19	0.19 – 18.63	0.9927
9-*Z*-*β*-carotene	0.129	0.93	0.93 – 18.63	0.9967
lycopene	0.005	0.09	0.09 – 9.31	0.9974

LoD, Limit of detection; LoQ, limit of quantification; r, correlation coefficient.

Table S2. Main dietary nutrient intake and food consumption by group.

	**Low F&V**		**High F&V**		***p*-value***
	**Low-to-Moderate fat**	**Very high fat**	**Low-to-Moderate fat**	**Very high fat**	
No. of subjects	59	58	60	53	
Mediterranean diet adherence score	7.29 ± 2.33	6.67 ± 2.56	10.0 ± 2.49	9.83 ± 2.79	< 0.001
Total energy, Kcal/day	1701 ± 511	2803 ± 420	2026 ± 373	3161 ± 356	< 0.001
Nutrient intake					
Carbohydrates, g/day	180 ± 77.7	256 ± 74.8	249 ± 66.7	324 ± 67.9	< 0.001
Fiber, g/day	15.5 ± 6.15	19.2 ± 4.79	35.3 ± 6.39	42.5 ± 8.87	< 0.001
Protein, g/day	70.8 ± 18.2	99.1 ± 19.6	89.8 ± 19.5	127 ± 21.7	< 0.001
Total fat, g/day	64.8 ± 15.3	140 ± 14.6	69.7 ± 12.6	142 ± 16.9	< 0.001
SFA, g/day	17.7 ± 4.44	36.9 ± 7.19	17.8 ± 4.66	34.5 ± 7.14	< 0.001
MUFA, g/day	32.4 ± 8.69	72.8 ± 11.0	33.7 ± 7.81	75.5 ± 12.4	< 0.001
PUFA, g/day	9.87 ± 3.56	22.5 ± 6.10	12.2 ± 3.98	25.3 ± 7.75	< 0.001
Cholesterol, mg/day	292 ± 105	437 ± 130	317 ± 97.1	459 ± 134	< 0.001
Alcohol, g/day	16.3 ± 20.9	17.3 ± 18.1	6.08 ± 11.1	11.6 ± 13.4	< 0.001
Food consumption, g/day					
F&V	278 ± 68.7	300 ± 52.7	1265 ± 299	1328 ± 270	< 0.001
Legumes	14.2 ± 7.95	18.3 ± 7.94	24.3 ± 16.4	30.4 ± 17.2	< 0.001
Cereals	140 ± 88.1	170 ± 85.3	129 ± 78.7	177 ± 82.4	0.005
Dairy	244 ± 113	360 ± 226	297 ± 213	380 ± 216	0.001
Meat	117 ± 44.9	151 ± 58.4	120 ± 48.0	171 ± 67.9	< 0.001
Fish	64.6 ± 38.5	92.6 ± 53.8	100 ± 48.0	136 ± 56.5	< 0.001
Nuts	5.11 ± 6.53	20.6 ± 21.4	10.5 ± 10.5	39.1 ± 30.7	< 0.001
Olive oil	23.2 ± 11.1	53.4 ± 15.5	24.0 ± 10.6	48.1 ± 16.4	< 0.001
Sunflower oil	1.40 ± 3.76	2.04 ± 5.72	0.91 ± 3.53	2.34 ± 7.22	0.455
Butter	0.75 ± 2.22	1.87 ± 4.77	0.56 ± 2.18	0.87 ± 2.08	0.092
Margarine	0.68 ± 1.65	1.38 ± 3.44	0.35 ± 0.98	1.34 ± 3.08	0.065
Pastries	18.4 ± 20.2	40.9 ± 43.6	14.6 ± 18.8	31.8 ± 32.1	< 0.001

SFA, saturated fatty acids; MUFA, monounsaturated fatty acids; PUFA, polyunsaturated fatty acids; F&V, fruit and vegetables.
Values are percentages for categorical variables and means ± SD for continuous variables.
* *P*-values were calculated by analysis of variance–one factor was used for continuous variables and the χ^2-test for categorical variables, $p < 0.05$.

Table S3. Differences in individual carotenoids plasma concentrations (µmol/L) between F&V consumption groups.

		High F&V vs. Low F&V	*p*-value	High F&V vs. Low F&V (low-to-moderate fat)	*p*-value	High F&V vs. Low F&V (high fat)	*p*-value
α-carotene	Median	0.19 vs. < 0.12		0.21 vs. < 0.12		0.14 vs. < 0.12	
	ß [CI]-model 1	0.15 [0.058; 0.24]	0.001	0.18 [0.052; 0.30]	0.005	0.11 [-0.017; 0.24]	0.088
	ß [CI]-model 2	0.11 [0.022; 0.20]	0.014	0.13 [0.007; 0.25]	0.038	0.088 [-0.039; 0.21]	0.173
	ß [CI]-model 3	0.10 [-0.006; 0.21]	0.064	0.094 [-0.048; 0.24]	0.197	0.045 [-0.1; 0.19]	0.553
β-carotene	Median	1.78 vs. < 0.02		2.55 vs. < 0.02		0.71 vs. < 0.02	
	ß [CI]-model 1	4.25 [2.27; 6.22]	< 0.001	4.97 [2.33; 7.61]	< 0.001	3.24 [0.40; 6.07]	0.025
	ß [CI]-model 2	3.76 [1.79; 5.73]	< 0.001	4.34 [1.71; 6.98]	0.001	2.93 [0.14; 5.71]	0.039
	ß [CI]-model 3	4.31 [1.98; 6.64]	< 0.001	4.52 [1.40; 7.64]	0.005	3.05 [-0.21; 6.31]	0.066
E-lycopene	Median	< 0.005 vs. < 0.005		< 0.09 vs. < 0.005		< 0.005 vs. < 0.005	
	ß [CI]-model 1	1.00 [-0.10; 2.10]	0.075	1.5 [-0.021; 3.03]	0.053	0.41 [-1.19; 2.00]	0.617
	ß [CI]-model 2	0.82 [-0.29; 1.93]	0.146	1.26 [-0.27; 2.80]	0.107	0.31 [-1.28; 1.89]	0.704
	ß [CI]-model 3	0.72 [-0.59; 2.02]	0.282	1.45 [-0.36; 3.26]	0.115	0.54 [-1.30; 2.37]	0.567
Z-lycopene	Median	< 0.005 vs. < 0.005		< 0.005 vs. < 0.005		< 0.005 vs. < 0.005	
	ß [CI]-model 1	0.63 [-0.41; 1.68]	0.233	1.14 [-0.30; 2.57]	0.120	0.042 [-1.48; 1.56]	0.957
	ß [CI]-model 2	0.48 [-0.57; 1.54]	0.368	0.93 [-0.52; 2.39]	0.207	-0.039 [-1.55; 1.47]	0.959
	ß [CI]-model 3	0.024 [-1.22; 1.27]	0.970	0.35 [-1.34; 2.03]	0.686	-0.58 [-2.33; 1.18]	0.518
Sum lycopene	Median	< 0.09 vs. < 0.09		0.29 vs. < 0.09		< 0.09 vs. < 0.09	
	ß [CI]-model 1	1.63 [-0.17; 3.43]	0.076	2.56 [0.07; 5.05]	0.044	0.55 [-2.06; 3.15]	0.681
	ß [CI]-model 2	1.35 [-0.47; 3.17]	0.145	2.18 [-0.33; 4.70]	0.089	0.39 [-2.20; 2.98]	0.768
	ß [CI]-model 3	0.94 [-1.21; 3.09]	0.393	2.01 [-0.94; 4.97]	0.182	0.30 [-2.71; 3.31]	0.845
astaxanthin	Median	0.44 vs. 0.35		0.42 vs. 0.31		0.48 vs. 0.36	
	ß [CI]-model 1	0.15 [0.038; 0.26]	0.009	0.18 [0.027; 0.34]	0.022	0.12 [-0.044; 0.28]	0.155
	ß [CI]-model 2	0.14 [0.027; 0.26]	0.016	0.17 [0.014; 0.33]	0.033	0.11 [-0.05; 0.27]	0.175
	ß [CI]-model 3	0.12 [-0.015; 0.25]	0.082	0.17 [-0.011; 0.35]	0.066	0.068 [-0.12; 0.25]	0.471
lutein	Median	< 0.88 vs. < 0.02		< 0.88 vs. < 0.88		< 0.88 vs. < 0.02	
	ß [CI]-model 1	0.64 [0.29; 0.99]	< 0.001	0.63 [0.16; 1.10]	0.009	0.65 [0.14; 1.16]	0.012
	ß [CI]-model 2	0.52 [0.18; 0.87]	0.003	0.48 [0.013; 0.94]	0.044	0.57 [0.073; 1.06]	0.025
	ß [CI]-model 3	0.48 [0.076; 0.88]	0.020	0.31 [-0.23; 0.84]	0.259	0.33 [-0.23; 0.90]	0.250
canthaxanthin	Median	0.16 vs. 0.18		0.18 vs. 0.18		0.15 vs. 0.17	
	ß [CI]-model 1	0.001 [-0.062; 0.064]	0.969	0.027 [-0.06; 0.11]	0.542	-0.028 [-0.12; 0.062]	0.538
	ß [CI]-model 2	-0.003 [-0.067; 0.062]	0.937	0.022 [-0.067; 0.11]	0.626	-0.031 [-0.12; 0.06]	0.505
	ß [CI]-model 3	0.039 [-0.037; 0.12]	0.314	0.023 [-0.079; 0.12]	0.663	-0.02 [-0.13; 0.085]	0.710
β-cryptoxanthin	Median	0.72 vs. 0.24		0.78 vs. 0.24		0.69 vs. 0.24	
	ß [CI]-model 1	0.58 [0.42; 0.75]	< 0.00001	0.70 [0.47; 0.93]	< 0.00001	0.45 [0.21; 0.69]	< 0.001
	ß [CI]-model 2	0.58 [0.41; 0.75]	< 0.00001	0.69 [0.46; 0.93]	< 0.00001	0.45 [0.21; 0.69]	< 0.001
	ß [CI]-model 3	0.60 [0.40; 0.81]	< 0.00001	0.70 [0.43; 0.97]	< 0.00001	0.44 [0.16; 0.72]	0.002

ß, difference between groups; CI, confidence interval.

Model 1— adjusted for age and sex. Model 2—adjusted for age, sex and physical activity. Model 3—adjusted for the variables used in model 2 plus energy intake, the modified Mediterranean diet adherence score (subtracting the questions regarding F&V, fat and wine) and alcohol consumption (g/day). *P*-values < 0.05 were considered significant.

Table S4. Differences in individual carotenoids plasma concentrations (μmol/L) between fat intake groups.

		High fat vs. Low-to-Moderate fat	p-value	High fat vs. Low-to-Moderate fat (low F&V)	p-value	High fat vs. Low-to-Moderate fat (high F&V)	p-value
α-carotene	Median	< 0.12 vs. 0.16		< 0.12 vs. < 0.12		0.14 vs. 0.21	
	ß [CI]-model 1	-0.049 [-0.14; 0.043]	0.295	-0.012 [-0.14; 0.12]	0.858	-0.076 [-0.20; 0.051]	0.241
	ß [CI]-model 2	-0.053 [-0.14; 0.035]	0.239	-0.027 [-0.15; 0.098]	0.671	-0.07 [-0.19; 0.053]	0.265
	ß [CI]-model 3	-0.13 [-0.28; 0.008]	0.064	-0.071 [-0.25; 0.11]	0.437	-0.12 [-0.30; 0.059]	0.189
β-carotene	Median	< 0.02 vs. 0.33		< 0.02 vs. < 0.02		0.71 vs. 2.55	
	ß [CI]-model 1	-1.87 [-3.87; 0.12]	0.066	-0.78 [-3.63; 2.07]	0.592	-2.51 [-5.1; 0.073]	0.057
	ß [CI]-model 2	-1.88 [-3.81; 0.051]	0.056	-0.97 [-3.76; 1.82]	0.496	-2.39 [-4.92; 0.15]	0.065
	ß [CI]-model 3	-3.70 [-6.85; -0.55]	0.021	-0.61 [-4.65; 3.43]	0.767	-2.08 [-5.89; 1.74]	0.287
E-lycopene	Median	< 0.005 vs. < 0.005		< 0.005 vs. < 0.005		< 0.005 vs. < 0.09	
	ß [CI]-model 1	-0.38 [-1.49; 0.73]	0.501	0.23 [-1.35; 1.80]	0.777	-0.87 [-2.41; 0.67]	0.269
	ß [CI]-model 2	-0.42 [-1.51; 0.67]	0.452	0.12 [-1.45; 1.68]	0.884	-0.84 [-2.37; 0.69]	0.281
	ß [CI]-model 3	0.30 [-1.45; 2.06]	0.734	1.39 [-0.90; 3.68]	0.235	0.47 [-1.72; 2.66]	0.672
Z-lycopene	Median	< 0.005 vs. < 0.005		< 0.005 vs. < 0.005		< 0.005 vs. < 0.005	
	ß [CI]-model 1	-0.29 [-1.34; 0.77]	0.595	0.30 [-1.19; 1.79]	0.694	-0.80 [-2.27; 0.68]	0.289
	ß [CI]-model 2	-0.31 [-1.35; 0.73]	0.559	0.21 [-1.27; 1.69]	0.781	-0.76 [-2.22; 0.70]	0.305
	ß [CI]-model 3	-0.31 [-1.97; 1.35]	0.718	0.14 [-2.00; 2.28]	0.896	-0.78 [-2.85; 1.28]	0.458
Sum lycopene	Median	< 0.09 vs. < 0.09		< 0.09 vs. < 0.09		< 0.09 vs. 0.29	
	ß [CI]-model 1	-0.59 [-2.40; 1.23]	0.526	0.52 [-2.05; 3.10]	0.691	-1.49 [-4.02; 1.03]	0.246
	ß [CI]-model 2	-0.65 [-2.44; 1.14]	0.478	0.35 [-2.21; 2.91]	0.79	-1.45 [-3.94; 1.05]	0.257
	ß [CI]-model 3	0.072 [-2.78; 2.93]	0.961	1.69 [-2.04; 5.41]	0.375	-0.029 [-3.59; 3.53]	0.987
astaxanthin	Median	0.37 vs. 0.38		0.36 vs. 0.31		0.48 vs. 0.42	
	ß [CI]-model 1	0.045 [-0.067; 0.16]	0.428	0.078 [-0.08; 0.24]	0.330	0.012 [-0.15; 0.17]	0.887
	ß [CI]-model 2	0.039 [-0.074; 0.15]	0.496	0.075 [-0.083; 0.23]	0.351	0.013 [-0.15; 0.17]	0.875
	ß [CI]-model 3	-0.059 [-0.23; 0.12]	0.511	0.056 [-0.16; 0.28]	0.617	-0.044 [-0.26; 0.18]	0.700
lutein	Median	< 0.88 vs. < 0.88		< 0.02 vs. < 0.88		< 0.88 vs. < 0.88	
	ß [CI]-model 1	-0.32 [-0.67; 0.037]	0.079	-0.33 [-0.83; 0.18]	0.204	-0.31 [-0.78; 0.17]	0.208
	ß [CI]-model 2	-0.33 [-0.67; 0.014]	0.060	-0.37 [-0.86; 0.12]	0.140	-0.28 [-0.74; 0.18]	0.233
	ß [CI]-model 3	-0.68 [-1.22; -0.15]	0.013	-0.52 [-1.21; 0.17]	0.139	-0.49 [-1.16; 0.17]	0.147
canthaxanthin	Median	0.15 vs. 0.18		0.17 vs. 0.18		0.15 vs. 0.18	
	ß [CI]-model 1	-0.041 [-0.10; 0.022]	0.206	-0.014 [-0.10; 0.074]	0.763	-0.069 [-0.16; 0.021]	0.132
	ß [CI]-model 2	-0.041 [-0.10; 0.022]	0.199	-0.015 [-0.10; 0.073]	0.731	-0.068 [-0.16; 0.021]	0.135
	ß [CI]-model 3	-0.12 [-0.22; -0.018]	0.020	-0.096 [-0.22; 0.029]	0.131	-0.14 [-0.27; -0.012]	0.032
β-cryptoxanthin	Median	0.38 vs. 0.51		0.24 vs. 0.24		0.69 vs. 0.78	
	ß [CI]-model 1	-0.18 [-0.36; -7e-05]	0.05	-0.043 [-0.28; 0.19]	0.720	-0.29 [-0.52; -0.058]	0.015
	ß [CI]-model 2	-0.19 [-0.37; -0.006]	0.042	-0.045 [-0.28; 0.19]	0.705	-0.29 [-0.52; -0.057]	0.015
	ß [CI]-model 3	-0.42 [-0.70; -0.13]	0.004	0.035 [-0.30; 0.37]	0.837	-0.23 [-0.56; 0.11]	0.186

ß, difference between groups; CI, confidence interval.

Model 1— adjusted for age and sex. Model 2—adjusted for age, sex and physical activity. Model 3—adjusted for the variables used in model 2 plus energy intake, the modified Mediterranean diet adherence score (subtracting the questions regarding F&V, fat and wine) and alcohol consumption (g/day). P-values < 0.05 were considered significant.

Table S5. Differences in individual carotenoids plasma concentrations (μmol/L) between extreme groups.

		Low-to-Moderate fat & high F&V vs. High fat & low F&V	*p*-value	High fat & high F&V vs. Low-to-Moderate fat & low F&V	*p*-value
α-carotene	Median	0.21 vs. < 0.12		0.14 vs. < 0.12	
	ß [CI]-model 1	0.19 [0.063; 0.31]	0.003	0.10 [-0.028; 0.23]	0.126
	ß [CI]-model 2	0.16 [0.035; 0.28]	0.012	0.061 [-0.066; 0.19]	0.348
	ß [CI]-model 3	0.16 [0.013; 0.32]	0.033	-0.026 [-0.26; 0.20]	0.825
β-carotene	Median	2.55 vs. < 0.02		0.71 vs. < 0.02	
	ß [CI]-model 1	5.75 [3.03; 8.48]	< 0.001	2.46 [-0.30; 5.22]	0.081
	ß [CI]-model 2	5.31 [2.63; 8.00]	< 0.001	1.96 [-0.78; 4.69]	0.161
	ß [CI]-model 3	5.13 [1.82; 8.43]	0.002	2.44 [-2.64; 7.53]	0.347
E-lycopene	Median	< 0.09 vs. < 0.005		< 0.005 vs. < 0.005	
	ß [CI]-model 1	1.28 [-0.24; 2.79]	0.098	0.63 [-0.97; 2.24]	0.438
	ß [CI]-model 2	1.15 [-0.36; 2.65]	0.136	0.42 [-1.18; 2.03]	0.605
	ß [CI]-model 3	0.062 [-1.79; 1.91]	0.948	1.93 [-1.00; 4.85]	0.197
Z-lycopene	Median	< 0.005 vs. < 0.005		< 0.005 vs. < 0.005	
	ß [CI]-model 1	0.84 [-0.60; 2.28]	0.253	0.34 [-1.18; 1.86]	0.660
	ß [CI]-model 2	0.72 [-0.71; 2.16]	0.323	0.17 [-1.36; 1.7]	0.827
	ß [CI]-model 3	0.20 [-1.55; 1.96]	0.820	-0.44 [-3.16; 2.29]	0.754
Sum lycopene	Median	0.29 vs. < 0.09		< 0.09 vs. < 0.09	
	ß [CI]-model 1	2.04 [-0.44; 4.52]	0.107	1.07 [-1.55; 3.69]	0.423
	ß [CI]-model 2	1.83 [-0.63; 4.30]	0.145	0.74 [-1.89; 3.36]	0.582
	ß [CI]-model 3	0.33 [-2.69; 3.35]	0.831	1.99 [-2.77; 6.74]	0.413
astaxanthin	Median	0.42 vs. 0.36		0.48 vs. 0.31	
	ß [CI]-model 1	0.11 [-0.051; 0.26]	0.187	0.20 [0.034; 0.36]	0.018
	ß [CI]-model 2	0.099 [-0.059; 0.26]	0.218	0.19 [0.023; 0.35]	0.025
	ß [CI]-model 3	0.11 [-0.075; 0.30]	0.242	0.12 [-0.16; 0.41]	0.395
lutein	Median	< 0.88 vs. < 0.02		< 0.88 vs. < 0.88	
	ß [CI]-model 1	0.96 [0.46; 1.46]	< 0.001	0.33 [-0.16; 0.81]	0.190

	ß [CI]-model 2	0.85 [0.37; 1.33]	< 0.001	0.20 [-0.28; 0.68]	0.413
	ß [CI]-model 3	0.83 [0.25; 1.40]	0.005	-0.19 [-1.05; 0.68]	0.675
canthaxanthin	Median	0.18 vs. 0.17		0.15 vs. 0.18	
	ß [CI]-model 1	0.041 [-0.047; 0.13]	0.363	-0.042 [-0.13; 0.048]	0.362
	ß [CI]-model 2	0.038 [-0.051; 0.13]	0.404	-0.046 [-0.14; 0.045]	0.321
	ß [CI]-model 3	0.12 [0.012; 0.23]	0.029	-0.12 [-0.28; 0.047]	0.162
β-cryptoxanthin	Median	0.78 vs. 0.24		0.69 vs. 0.24	
	ß [CI]-model 1	0.74 [0.51; 0.97]	< 0.00001	0.41 [0.17; 0.64]	< 0.001
	ß [CI]-model 2	0.74 [0.51; 0.97]	< 0.00001	0.4 [0.16; 0.64]	0.001
	ß [CI]-model 3	0.66 [0.38; 0.95]	< 0.00001	0.47 [0.038; 0.91]	0.033

ß, difference between groups; CI, confidence interval.

Model 1— adjusted for age and sex. Model 2—adjusted for age, sex and physical activity. Model 3—adjusted for the variables used in model 2 plus energy intake, the modified Mediterranean diet adherence score (subtracting the questions regarding F&V, fat and wine) and alcohol consumption (g/day). *P*-values < 0.05 were considered significant.
